# Barriers and strategies for implementing community-based interventions with minority elders: positive minds-strong bodies

**DOI:** 10.1186/s43058-020-00034-4

**Published:** 2020-04-30

**Authors:** Thalia Porteny, Margarita Alegría, Paola del Cueto, Larimar Fuentes, Sheri Lapatin Markle, Amanda NeMoyer, Giselle K. Perez

**Affiliations:** 1 Interfaculty Initiative in Health Policy, Graduate School of Arts and Science, Harvard University, 1350 Massachusetts Avenue, Suite 350, Cambridge, MA 02138-3654 USA; 2 Disparities Research Unit, Department of Medicine, Massachusetts General Hospital, 50 Staniford Street, Boston, MA 02114 USA; 3 Department of Psychiatry, Harvard Medical School, 401 Park Drive, Boston, MA 02215 USA; 4 Department of Health Care Policy, Harvard Medical School, 180 Longwood Avenue, Boston, MA 02115 USA; 5 Health Policy Research Center at the Mongan Institute, Massachusetts General Hospital, 100 Cambridge Street, Boston, MA 02114 USA

**Keywords:** Racial/ethnic minority elders, Depression, Anxiety, Disability, CHW, Immigrants

## Abstract

**Background:**

By 2040, one out of three older adults in the USA are expected to belong to a racial/ethnic minority group. This population has an increased risk of mental and physical disability with significant barriers to access care. Community-based organizations (CBOs) often provide programming to serve minority and immigrant elders. Limited resources and other barriers such as lack of trained staff make it difficult to implement evidence-based interventions (EBIs) in CBOs for long-term adoption. Yet little is known about what factors can facilitate adoption of EBIs in CBOs serving minority elders.

**Methods:**

Positive-Minds–Strong Bodies (PM-SB), an evidence-based intervention offered in four languages, aims to reduce mental and physical disability for minority and immigrant elders through the efforts of community health workers and exercise trainers. The intervention consists of cognitive behavior therapy and exercise training sessions delivered over 6 months. During a recent clinical trial of this intervention, we elicited feedback from CBO staff to determine how best to facilitate the implementation and long-term sustainability of PM-SB within their agencies. We surveyed 30 CBO staff members, held four focus groups, and conducted 20 in-depth interviews to examine staff perspectives and to reveal factors or changes needed to facilitate long-term adoption in prospective CBOs.

**Results:**

Participants reported that staff motivation and implementation could be improved through the following changes: increasing patient compensation for treatment sessions, decreasing levels of organizational accountability, and reducing staff demands embedded in the intervention. Although most staff perceived that PM-SB improved their agency’s ability to address the health and well-being of elders, capacity-building strategies such as a “train-the-trainer” initiative were identified as priorities to address staff turnover for sustainability. Adapting the intervention to get financial reimbursement also emerged as vital.

**Conclusions:**

Augmenting financial incentives, streamlining procedures, and simplifying staff accountability were suggested strategies for facilitating the transition from a disability prevention clinical trial in minority and immigrant elders to a scalable implementation in routine services at CBOs.

**Trial registration:**

ClinicalTrials.gov, NCT02317432.

Contributions to the literature

- This research examines staff’s perceived barriers and facilitators for implementing an evidence-based mental health and physical strengthening program for minority elders within community-based organizations.
- The Interactive systems framework for dissemination and implementation was used to assess staff’s self-perceived individual, organizational, and community level capacities for the adoption and implementation of the intervention.
- Staff-recommended adaptations for implementing community-based interventions can inform policies and programs on how to improve staff adoption of evidence-based programs in community-based organizations.
- Project staff identified factors that can help transition disability prevention programs from research to local implementation, a novel approach.

## Background

Minority elders represent a rapidly increasing sector of the aging population [1–3]. In the USA, one in every four elders (i.e., people over 65) are part of a racial/ethnic minority group, and by 2040 that rate will be one in three [4]. Elevated symptoms of depression [5–7], anxiety [8, 9], physical disability [10, 11], and dementia [12] are common in older adults, especially in minority elders [3]. Yet, there are significant barriers to obtaining preventative treatment [9]. These symptoms are often unrecognized by health providers [13, 14] and treated as a normal component of aging [15] and the accompanying loss of social ties [16]. Even when symptoms are recognized, elders may not access services because of a lack of providers who can serve linguistic minorities, transportation difficulties, and limited knowledge of where to go [17]. When these conditions are untreated, they lead to substantial incapacity and increased risk of disability [5, 18, 19].

Community-based organizations (CBOs) are vital channels that offer elder minorities easy access to participate in community activities with trusted personnel, preventing the loss of social ties, and the lack of recognition of mood symptoms [20, 21]. Due to their potential reach and impact, these agencies are also crucial platforms for clinical trials that aim to design prevention and evidence-based intervention programs (EBIs) for mental health and physical strengthening in elder minorities [22–25]. Yet, funding in many CBOs is limited and organizational barriers can hinder the transition of an EBI within a disability prevention clinical trial to an EBI that is formally embedded into routine care [25]. In the current study, implementation includes “the use of strategies to adopt evidence-based health interventions and change practice patterns within specific settings” [26, 27]. Given the widely documented financial and structural barriers, CBOs must often adapt evidence-based programs when employing them in “real world” settings [28]. Here, we define adaptations as modifications that can be made to the EBI in the process of its implementation and adoption as the integration of the EBI into practice [29]. Thus, in this context, interventions must go through an adaptation process for them to be adopted within the reality and constraints of CBOs [30, 31]. Our focus here is mainly on adaptations needed for implementation and not adaptations made to the actual intervention (e.g., content, materials, and delivery).

Little research has examined challenges to sustained EBI implementation within a CBO. Given the unique needs of minority elders, it is particularly important to investigate strategies to overcome these barriers. Understanding and incorporating the perspectives of CBO staff can fast-track site adaptations needed to successfully embed EBI into CBOs by identifying individual and system-level barriers and facilitators [32–34]. The present study thus examines CBO staff perspectives of positive minds–strong bodies (PM-SB) following their participation in a multi-site clinical trial examining program effectiveness [35]. PM-SB aims to reduce mental and physical disability for English, Spanish, Mandarin, and Cantonese speaking older adults (60 + years) who present with elevated depression and anxiety disorder symptoms and functional deterioration. The intervention consists of ten individual sessions of cognitive-behavioral therapy delivered by community health workers (CHWs) embedded within CBOs. These sessions are concurrently offered with 36 group sessions of strengthening exercise training provided by exercise trainers (ET). Study research assistants (RAs) recruit participants and collect participant progress data. Additionally, the CBO has site-leaders and coordinators that supervise and support outreach and implementation initiatives. Trial results, described elsewhere [35], show effectiveness of the PM-SB intervention after 6 months, as participants demonstrated improved physical functioning and reduced mental health symptoms.

The clinical trial provided a unique opportunity to investigate factors that will facilitate PM-SB’s transition from research protocol to a scalable program for elder minorities served by CBOs. In the current study, we addressed two main questions: first, do CBO staff perceive the clinical trial as building their capacity to implement PM-SB in CBOs? Second, what do CBO staff identify as barriers to implementation after the clinical trial ends, and what strategies can be used to overcome them? Findings highlight recommendations to ensure long-term PM-SB adoption by CBOs in the future and, more broadly, help improve EBI implementation in CBOs.

## Methods

### Evaluation framework

Consistent with previous work that investigates staff perspectives on how to close the “research-to-practice implementation gap,” we used the interactive systems framework (ISF) for dissemination and implementation as a helpful lens to guide analysis [34, 36]. This framework posits that three systems interact at different levels to bridge research and practice: delivery systems, support systems, and synthesis and translation systems. As applied to this study, CBOs are the *delivery system*, and minority elders are the primary recipients of the services delivered. Since PM-SB is transitioning from a trial to an EBI, the *support systems* that provide training and technical assistance will no longer be the academic partners, but rather the staff that implements the PM-SB [25, 36]. Academic institutions are working in tandem with staff that implements PM-SB to detect strategies that can overcome implementation barriers, translating them into research findings and tools to improve adoption; thereby functioning as a *synthesis and translation system* that goes back to the *delivery.*

The ISF was applied to categorize factors that may facilitate or hinder the implementation of PM-SB within the following three levels:

- Individual capacity: (e.g., staff’s perceived capacity to implement the intervention)
- Organizational capacity: (e.g., organizational climate, structure, and resource availability to deploy the EBI)
- Community capacity (e.g., leadership within the community as well as connections within and outside the community, including institutions)

### Design

We used a mixed-methods approach with a specific structure, function, and process that is consistent with implementation research [37]. We simultaneously collected data from surveys (quantitative component), focus groups, and in-depth interviews (qualitative components), giving equal weight to both types of data. This structure converges data about staff perceptions of the trial’s ability to build capacity for implementation. Additionally, qualitative methods were applied to elaborate on strategies to overcome implementation barriers at the individual, organizational, and community levels. Through this process, we triangulated the multiple data sources to validate the consistency of our findings [37, 38]. A brief summary of the study methods is presented in Table 1.

**Table 1:** Summary of methods

	Survey (quantitative data) *N* = 30	Focus groups (qualitative data) *N* = 30	In-depth interview (qualitative data) *N* = 20
Participants	CHW, ET, RAs, and site leaders that participated in stakeholder meeting		Subpopulation of CHW, ET, RAs, and site leaders that participated in stakeholder meeting and volunteered for follow-up interviews
Procedure	Anonymous paper-based survey Duration: ~ 15–20 min	Two-step group discussion guided by questionnaire 1. Small focus groups based on the role (i.e., site leader, CHW, ET, RA) Duration: ~ 45 min 2. Large group discussion Duration: ~ 35 min	In-depth semi-structured interview based on an interview guide Duration: ~ 20–30 min
Individual capacity	Self-reported knowledge about the project (how intervention is delivered) Self-reported skills (adequate training) Resources (adequate time) Motivation (intervention is useful to me and agency) Awareness (agency’s involvement improves well-being of population)	N/A	Would you be willing to continue to work on [exercise training] once partnering organizations are not involved?
Overall barriers	N/A	What are the greatest barriers to maintain the program?	What are some of the issues that may arise for you after the trial ends to be able to continue to implement PM-SB?
Overall facilitators	N/A	What supports would you need to maintain the program?	Is there anything you think [CHW] could do now to help ensure PM-SB continues to be implemented after the trial ends?
Emerging themes	Purpose and goals of project are clear Intervention is perceived as impactful Adequate training and understanding of responsibilities but not enough time to fulfill them	Staff turnover is a barrier to implementation Restructuring was disruptive leading to feelings of burnout Trainings and intervention are successful. Capacity building strategies for long-term adoption should include adapting tools Obtaining funds is critical for continuation of intervention	Staff turnover is a barrier to implementation, burnout and compensation are related issues Trainings can be adapted to include a train the trainer model and continue to include methodologies that utilize: technology, role-play and cultural adaptations Funding strategies to overcome financial obstacles such as reimbursing through Medicare and Medicaid should be implemented

### Quantitative component

#### Participants

CBO staff were invited to participate in the current study during a PM-SB stakeholder meeting that took place in January 2018 in Boston, Massachusetts. The meeting included staff from the six implementing CBOs (three from Boston and one each from New York, Florida, and Puerto Rico). PM-SB staff implementing the program (*n* = 30) were surveyed. They included eight site leaders/coordinators, seven CHWs, four ETs, eight RAs, and five other research team members. See Table 2 for more details of participants’ characteristics.

**Table 2:** Participant characteristics for survey and focus groups during stakeholder meeting

	# of participants (***N*** = 30)
**Role**^**a**^	
Site leader	8
Community health worker	7
Exercise trainer	4
Research assistant	8
Other research team member	5
**Length of time at current agency**	
Less than 1 year	8
1–3 years	17
3–5 years	2
5–9 years	1
10 + years	2
**Prior experience with research**	
Yes	16

Note. Table adapted from 2018 PM-SB Partnership evaluation

^a^One participant indicated more than one role (one CHW/RA/other research team member)

#### Procedure

We investigated the staff’s perceived individual capacity (i.e., awareness, knowledge, skills, self-efficacy, and motivation) [39] to provide the PM-SB intervention because it is hypothesized to affect future adoption of EBIs and the success of the implementation. Participants were invited to respond to an anonymous paper-based survey during a stakeholder meeting that assesses the following domains: (1) “understanding the purpose of the study,” (2) “roles and responsibilities,” and (3) “perceived impact.” Measures are adapted from previous studies deriving instruments to assess the capacity of community-based initiatives [40, 41].

Participants were instructed to rate their agreement with a series of 16 statements from the three domains on a 5-point Likert scale (1 = strongly agree to 5 = strongly disagree). The “understanding the purpose of the study” domain (5 items) asks participants to rate their self-reported knowledge about the project (e.g., “I understand how the intervention can be delivered in a community-based organization”). The “roles and responsibilities” domain (5 items) asks questions to analyze self-reported skills (e.g., “I have adequate training to fulfill my responsibilities”) and resources (e.g., “I have adequate time to fulfill my responsibilities”). Lastly, the “perceived impact” domain (6 items) examines staff’s motivations (e.g., “I am confident this intervention is useful to me and my agency”) and awareness (e.g., “My agency’s involvement will improve our ability to address the health and well-being of elders with disabilities”). We calculated descriptive statistics for each item. The results are reported using the median and the interquartile range (IQR)—the range between the 25th–75th percentile. Additionally, we present the percentage of participants that agreed or strongly agreed with each statement.

### Qualitative component

#### Focus groups

Following the paper-based survey, participants took part in small and large focus groups. Semi-structured discussion guides were designed by the study leaders to understand barriers to long-term program implementation at the individual, organizational, and community levels, and strategies to overcome those barriers. The main questions in the focus group discussions were “Do you see your organization maintaining this program?” “What supports would you need to maintain the program?” “What are the greatest barriers to maintaining the program?”

To discuss specific PM-SB role-based issues, individuals were divided into small focus groups based on their roles (e.g., CHW, physical trainer, site leaders) for approximately 45 min. Then participants took part in a large group discussion (35 min.) to share feedback from their small groups. This two-step process was designed to allow participants to first discuss concerns related to their specific roles and to ensure that all had a chance to speak before delving into a wider discussion. All focus group data were recorded by a designated note-taker. All notes were reviewed and analyzed by an independent consultant team that summarized the responses.

#### In-depth interviews

After the focus group discussions, 20 participants volunteered to participate in additional individual in-depth interviews, including six site leaders/program coordinators, five CHWs, five ETs, and four RAs (see Table 3 for more detailed characteristics). The primary researcher of this paper conducted 20- to 30-min individual semi-structured interviews in person (*n* = 8) and by phone (*n* = 12).

**Table 3:** Participant characteristics for in-depth interviews

Participants	# of participants (*N* = 20)
Site leaders/program coordinator	6
CHWs	5
Exercise trainers	5
Research assistants	4
Demographics	
Ethnicity	
Asian	10
Latino	9
White	1
Sex	
Female	15
Male	5

The in-depth interview guide includes four domains: recommendations for adapting the support system at the organizational level (e.g., “What are important steps to ensure staff is trained correctly once partnering organizations are not involved?”), staff’s individual perceived capacity (e.g., “Would you be willing to continue to work on [exercise training] once partnering research organizations are not involved?”), overall barriers (e.g., “What are some issues that may arise for you after the trial ends to be able to continue to implement PM-SB?”), and recommended strategies to overcome barriers (e.g., “Is there anything you think [CHWs] could do now to help ensure PM-SB continues to be implemented after the trial ends?”).

All domains included questions tailored to the role and experiences of each participant, with corresponding probes. For example, CHWs were presented with questions specific to CHW training. The interviews were audio-taped and transcribed verbatim by two researchers, stripping all identifiers. One interview was translated from Spanish to English by the bilingual interviewer for analysis purposes.

Two researchers reviewed the transcripts to identify themes and developed a coding framework. The same researchers independently performed open coding on the first two transcripts using a descriptive coding technique, where each emerging data element was assigned a code and included in a codebook. The codebook was updated and amended as descriptive coding continued and codes emerged. The two researchers met regularly to discuss the consistency of coding, possible new codes, and any coding challenges. Discrepancies in coding were resolved through consensus discussions between the two researchers. To ensure inter-coder reliability, a 3rd coder was included in four randomly selected interviews to discuss discrepancies and finalize the coding framework.

Analysis was then followed by two levels of thematic coding. The first level of thematic coding focused on sorting concepts into barriers and strategies to overcome barriers of PM-SB adoption. The second level of thematic analysis focused on axial coding by identifying and classifying concepts into themes, which had not been previously identified, and subthemes to better understand staff’s perceptions of adaptations to the PM-SB that would facilitate adoption. Thematic axial coding decisions were discussed among the research team. We used the qualitative research software Dedoose [42] to help guide the analysis.

## Results

### Quantitative analysis

Table 4 presents results from the paper-based anonymous survey to investigate staff’s individual capacity (i.e., knowledge about the intervention, skills, self-efficacy, motivation, and awareness about the impact of the intervention) as well as their perceptions of organizational and community level capacity through the different ISF systems. We found a high level of self-reported individual capacity in conducting the intervention and low heterogeneity, indicated by the percentage of respondents that strongly agree or agree with these statements (1 or 2 on Likert scale). Overall, at least 97% of participants understood the purpose of the intervention, the research associated with the intervention, and how PM-SB augments their capacity to serve elderly minorities. Nonetheless, 13% did not agree they understood (3–5 on Likert scale) how PM-SB may be implemented in community-based settings, showing at least some perceived barriers in delivery.

**Table 4:** Survey responses

Domains and questions	Median	IQR	% Agree
Purpose and goals			
I understand the goals of the project	1	1	100
I understand the purpose of delivering the intervention through a research study	1	1	100
I understand how the intervention can be implemented in a community-based organization	2	1	87
I think the outcomes that we hope to see for people involved in the intervention are appropriate	1	1	97
My agency’s involvement in this project is improving our capacity to serve elders in our community	1	1	97
Roles and responsibilities			
I have a clear understanding of my responsibilities on the project	2	1	97
I believe my responsibilities are appropriate	1	1	87
I have adequate training to fulfill my responsibilities	1	1	100
I have adequate time to fulfill my responsibilities	2	1.25	73
There are systems in place to ensure that all partners are fulfilling their responsibilities in a timely manner	2	1.25	77
I know whom to contact when I have a question about the project	1	1	93
Perceived impact			
My agency’s involvement will improve our ability to address the health and well-being of elders with disabilities	1	1	90
The project has increased the capacity of staff at my agency to meet the complex needs of elders with disabilities	2	1	80
Research activities help us understand the effectiveness of the intervention	2	1	97
The benefits of being involved in this project are greater than the challenges	1	1	90
I am confident that his intervention is useful to me and my agency	1	1	93

Note. Likert Scale (from 1 to 5: 1 being strongly agree and 5 being strongly disagree with a do not know option

There is some variation in staff’s responses to the “roles and responsibilities” domain. All participants (100%) believe they had sufficient training to meet responsibilities and 97% say they understood their responsibility, which portrays good capacity at the individual level and a satisfactory support system. Yet, 73% of participants indicated they have adequate time to fulfill responsibilities, and 77% said systems are in place to ensure partners fulfilled responsibilities. This variation shows some limitations in the delivery system at the organizational level.

Lastly, results show strong agreement and low heterogeneity with each of the perceived impact statements, indicating high motivation and awareness of the reach of the intervention. For example, 97% of participants agreed that research activities helped them understand the effectiveness of the intervention. This shows acceptability for the synthesis and translation systems. Additionally, 93% said that the benefits outweighed the challenges. Notably, 20% of respondents disagreed that the project has increased the capacity of staff to meet the needs of elders with disabilities, once again showing some limitations in the delivery system at the organizational level.

### Qualitative analysis

The thematic analysis findings from the in-depth interviews and focus groups validate quantitative findings. From participants’ responses, we identified the following barriers in the delivery and support systems at the organizational level: (1) staff turnover was an obstacle to meet the goals of the intervention, (2) staff time was not enough to fulfill their responsibilities, and (3) restructuring at sites disrupted teams. Participants recommended several strategies to overcome these barriers in EBI implementation: (1) ensuring CBOs’ ability to provide effective training and (2) securing funding. Overall, staff perceived the intervention to be effective at enhancing the lives of elder minorities because staff sensed an improvement in the elders’ moods and physical functioning. Participants reported that the intervention should continue and perceived their agencies would keep the program going, provided there was funding within CBOs to sustain it. See Table 5 for a summary of focus group discussions and Table 6 for a summary of thematic results from the in-depth interviews.

**Table 5:** Summary of barriers and facilitators from focus group discussions

ISF construct		
	Barriers	
**Delivery system**		
Organizational level	Staff turnover	Emerged due to restructuring in MGH and NYU sites → Turnover in site leaders is particularly disruptive. CHW feel pressure to deliver intervention before training is done. Not enough staff to implement intervention. Turnover leads to challenge in keeping CBO staff engaged. Balancing out labor demands with other full-time responsibilities in agency → burnout.
	**Facilitators**	
**Support system**		
Organizational level	Training	More guidelines regarding intervention adaptation and maintenance. Academic partners should provide additional training on adapting intervention for long-term adoption. Training would need to focus on how to maintain intervention fidelity while limiting data collection to only what is essential.
Organizational and community level	Funding	Using data for funding applications. Set aside time. Keep in mind different needs of sites. Research staff may be able to provide some support in future after grant ends.

**Table 6:** Summary of thematic analysis

ISF construct	Theme	Subtheme	Illustrative quotes
**Delivery system**			
**Organizational level**	Staff-turnover is an obstacle to meet goals of intervention	Staff perceive they should be paid more for the time commitment	*“I’m going after tomorrow... but pay the exercise trainers more because the rate I get is quite a bit lower than I would get as a personal trainer at the gym.”* *“[Being an RA] has been a great experience but I’m not getting paid to do this. Then I was given more tasks and things to do.”*
		Difficult to find motivated and qualified staff	*“Just motivating people in my position is very hard… it is very hard to motivate either the training of the CHW or the RAs the environment we have right now is uncomfortable.”* *“[there is a] lack of support from my supervisor and lack of interest, lack of commitment and lack of understanding of the trial.”*
**Support system**			
**Organizational level**	Ensuring CBOs ability to provide effective capacity-building	Identifying individuals in agency who are motivated and can be trained to train	*“It is important to find someone that is serious about being part of the program, understands it’s goals… has been in the organization for a longer period.”* *“We need people that are serious about being part of the program, because it helps to be invested in it. To understand what are the goals, the focus, and be mindful of the core concepts.”*
		Including technology	*“It would be easier if [CHWs] have recordings of sessions to practice before their initial training.”* *“Include videos for the exercise trainers so they learn the exercise before, but I think we still need to make sure the person is doing it correctly in person.”*
		More roleplay (CHW)	*“More roleplays would be good to help people practice. They need to practice more.”* *“Sometimes [CHWs] get stuck so someone else can help clarify the situations and concepts so they deliver more eloquently if they practice in roleplays.”* *“More roleplays would be good to make sure they understand the mindfulness CBT component, which is crucial.”*
		Cultural adjustments	*“Since Cantonese is a spoken language, the questions we have to read in Cantonese sound mechanic, so more flexibility in the interpretations.”* *“Let them talk about cultural stigma in the sessions because [Asian elders] come with a barrier to participate and if they talk about it, they feel more empowered and accepted.”*
	Having strategies to overcome financial obstacles	More flexibility with managing funds to adapt to local needs and improve compensation	*“There needs to be more flexibility in terms of supporting staff and understanding the real costs of the intervention at the center because that would allow us to figure out how much it really costs the organization to staff someone onboard.”*
**Community level**		Applying for other grants	*“I consider each organization should look for their own grants.”* *“I think we need to work to find sources of funding and write grants with help of partnering organizations.”* *“There is potential for other grants… or test a more adapted version of the [intervention].”* *“If [partnering organizations] can prove that [PM-SB] works and people are going to get better and benefit from this, then I think that’s enough to continue the intervention [through grants].”*
		Obtaining funds from the government or private sector to provide reimbursements	*“I think about Medicare and some are Medicare/Medicaid so there could be a reimbursement for prevention…Or United Healthcare reimburses local YMCAs for people they lift into diabetes prevention so maybe if we enroll a certain number of Medicare or Medicaid participants, [agencies] will get reimbursed at a certain level.”* *“The council in aging is connected to other senior centers so seeing how they could help out in terms of funding.”* *“We are working with a growing population, so I think the mayor of [site] would be interested in supporting the project and providing funding.”*

### Barriers in the delivery and support systems at the organizational level

#### Staff turnover as obstacle to meet the goals of the intervention

The main barrier that emerged in both focus groups and in-depth interviews across all roles was staff turnover. Within the theme of staff turnover, compensation and motivation were contributing factors that impacted staff’s roles and responsibilities. Some CBOs are not able to pay staff more than their full-time base salary within the CBO due federal contracts. Other CBOs recruit university students or other professionals as volunteers.

In both the focus groups and in-depth interviews, personnel stated that the high turnover rate within CBOs led to some difficulties with keeping CBO staff engaged with the project. They also voiced issues related to compensation, such as payment delays. During the in-depth interviews, some staff reiterated this issue. Additionally, ETs perceived compensation as lower ($40 a session) than what they would otherwise make in a gym, which they identified as the main reason for staff turnover*.*

#### Limited staff time to fulfill their responsibilities

In both focus groups and in-depth interviews, the staff stated that they struggled to figure out how to meet the demands of the project while balancing their other full-time job responsibilities. For the most part, their full-time responsibilities remained the same and the intervention is “added on” to their existing work, leading to burnout. For example, community-based RAs noted during the focus groups that it is challenging for them to conduct outreach work within the community on top of their other responsibilities. CHWs in focus groups stated that personnel shortages created an overload with attending the project cases. They usually had to complete two cases per CHW. They reported that completing more participant sessions are time- and labor-intensive when added to their full-time job. Site leaders also explained that the supervisory responsibilities are more than what they originally expected.

#### Restructuring at sites disrupts teams

During the focus group discussion, respondents indicated that challenges emerged due to significant restructuring within the sites, changes in site leadership, the withdrawal of existing CBO sites, and the recruitment of new CBO sites, as well as turnover within the academic and CBO teams. This barrier seemed to specifically affect RAs and CHWs. The RAs explained that the turnover in site leaders is particularly disruptive because they play a very significant role in the execution of the study. For example, RAs in one site explained experiencing burnout due to being asked to perform tasks that are not part of their role because of site leader turnover. New site leaders were less familiar with the division of roles on the project and there were not enough RAs to do the additional work (e.g., conducting baseline interviews on minority elders) that are assigned to them. RAs in focus groups also discussed how the turnover in academic research staff makes it challenging to fully support external CBO staff. Site leaders and CHWs explained feeling pressured to have new CHWs start delivering the intervention before they received enough training or developed enough confidence, in response to CHWs leaving an agency in the middle of the trial.

### Strategies to overcome barriers

#### Ensuring CBOs’ ability to provide effective training

During the focus groups, participants noted that current trainings were effective for the roles that PM-SB requires. Moving forward in the implementation stage, adaptations to the training of new staff are needed to ensure the intervention continues. More guidelines in an adapted manual regarding intervention adaptation and maintenance need to be developed as well as some additional training from the academic partners for long term-adoption. Participants explained that any future training would need to focus on how to maintain intervention fidelity. At the same time, they suggested limiting data collection to only what is essential.

In-depth interview participants perceived using a “train-the-trainer” model as a capacity-building priority, whereby identifying committed staff and teaching them to train could be a useful strategy]. Staff emphasized the importance of infrastructure in place to support ongoing training. They suggested that it is crucial to identify staff that are able and willing to train new staff once the trial ends. Being a staff member who has implemented the program for an extended period of 6 months or more and motivated about the project are considered factors needed for the staff who would train others.

During the in-depth interview, the staff suggested different strategies to ensure they are trained correctly when PM-SB is adopted. ETs recommended continuing to conduct supervision and fidelity checks through the current method, recording a strong bodies session and sending it to the supervisor. They also stated that having initial face-to-face training, which was a typical practice for the trial, is important to adequately conduct the exercises. ETs identified receiving training in a bigger group as a viable way to cut down costs while maintaining fidelity. CHWs also suggested continuing to include technology by audio recording and evaluating training sessions before the actual training. They believe this is useful to prepare staff that should be maintained during the implementation stage. Another strategy CHWs identified as helpful is continuing to include roleplays during the face-to-face training because it allows more practice before delivering sessions. CHWs that implement positive minds in the elder Asian population also suggested further cultural adaptations as a strategy to address cultural barriers of communication, such as including a session with PM-SB participants to talk about how social stigma affects mental health-seeking. They reiterated the importance of ensuring all materials to be culturally adapted for the target populations.

#### Securing funding

Having strategies to overcome financial obstacles was also a central theme that emerged during focus group discussions and in-depth interviews. In the focus groups, participants explained that applying for additional funding would be essential to maintain the program in CBOs. In Puerto Rico, they reported the need for more funding for transportation of participants and staff, as well as for training of additional CHWs to expand delivery of the intervention. Site leaders in Boston and New York also highlighted the need to fund transportation, explaining that extreme weather means they increasingly go to participants’ homes to conduct exercise sessions.

During the in-depth interviews, some participants suggested designing mechanisms for reimbursement from Medicare and Medicaid. They noted that many participants qualify for Medicare, and some are considered “duals” because they have both Medicaid and Medicare due to disability and being over 65 years of age. A related recommendation is to obtain reimbursement through insurance companies. Other recommendations include engaging local actors, such as the town mayor who might be interested in supporting the project and providing resources. Participants stated that even though the trial will end, partnering organizations could play an important role in securing funds. Their involvement in providing evidence of the intervention’s effectiveness and engaging stakeholders is perceived as a facilitator to receive additional funds since it gives CBOs an institutional backbone. Many staff believe that applying for grants is a feasible way to obtain funds and suggested continuing to work with partnering organizations to develop those grants.

## Discussion

This is one of the few studies that focus on staff perceptions of adaptations necessary to successfully implement an EBI in CBOs [20, 25, 32, 43]. To our knowledge, this study is among the first to focus on staff perceptions of changes needed to effectively implement an EBI for disability prevention in minority elders after a trial comes to an end. The qualitative methods allowed us to validate and elaborate on the quantitative findings, which was especially useful for confirming that staff felt they were skilled and highly capable of delivering the intervention. We also found overall acceptance and interest in implementing PM-SB, yet the mixed methods approach confirmed that challenges remain, mainly at the organizational level in the delivery system (e.g., limited time to perform responsibilities) and support system (e.g., restructuring roles at sites and academic centers), related to staff-turnover, renumeration, and burnout. Our results are consistent with challenges faced by other CBOs during EBI implementation [20, 25] and shed light on broader financial and staff-related issues that affect CBOs in low-resource settings. Staff recommendations for overcoming these barriers in implementation primarily involved the support system at both the organizational and community levels (e.g., training and insurance reimbursement opportunities). We focus our discussion on these issues and potential innovative solutions that can be integrated for the adoption of EBIs within CBOs in low-resource settings.

Consistent with previous research, our findings indicate the need to improve the delivery and support system at the organizational level, such as preventing staff-turnover by clarifying staff responsibilities and providing a supportive environment [44, 45]. Existing research on intervention programs implemented in CBOs suggests that not seeing a clear path towards career advancement is a common reason for turnover in community agencies [45]. In particular, young age and higher education along with job burnout are predictors of leaving the job altogether [46]. To counter this issue, reducing role ambiguity [47] and creating job sequences that lead to promotion can be better systematized in CBOs [45]. For example, intermediary positions such as leaders of particular roles (i.e., CHWs) or leaders of training can build upper-level positions that motivate staff to remain in their jobs [45]. These roles can be designed to increase individual employee autonomy and involvement in decision-making that can impact job satisfaction [48]. Developing a culture that rewards mentorship and efforts by the staff when participants have positive results also increases retention [46]. Further, using supervisors to actively participate in reflecting, focus, and decision-making about strategies and cases fosters a supportive environment and prevents burnout [49]. Other useful burnout prevention practices for staff in CBOs include time off from work and peer-networks, among others [48, 50, 51].

Our findings additionally suggest that integrating a “train the trainer” model with adaptations to the existing training tools and methods is perceived by PM-SB staff as a viable step to adapt PM-SB to widespread implementation, enhancing chances of adoption. Other programs that have used this model in CBOs have been shown to improve the psychosocial and physiological health status of the target population [52]. Evidence also suggests that a “train the trainer” model helps foster continuity, builds capacity at the local level, and enhances scalability [53]. Nonetheless, there are other factors that would need to be defined as the trial transitions to implementation, such as establishing what agency or entity would be in charge of supervising trainings and ensuring staff is sufficiently trained [54]. This is especially important if the scalable implementation of an EBI includes paraprofessionals as providers, such as university students. Having sufficient supervision and professional guidance is crucial since paraprofessional staff might not have the background training to deal with sensitive mental health situations such as suicide risk [54].

Our findings show variability between how CBOs could maintain fiscal and structural health and implement the program effectively. An illustrative example we found is the differences in how agencies compensated staff. Although funding for roles came from a research grant, this compensation was implemented differently in the organizations given local funding guidelines or restrictions. This is consistent with the literature in the sense that fiscal and structural health of a CBO impacts EBI implementation [24]. However, this finding sheds light on the importance of identifying financial structures for providing these types of health prevention services. CBOs need funding to ensure they can compensate workers adequately for EBI roles when they are combined with other funding streams for ongoing initiatives. As CBOs are becoming important providers for prevention programs, more community and agency assessment tools are needed to identify CBOs that can be effective as EBI providers [22].

Our study contributes to the literature in many ways. Staff offered helpful insight regarding strategies to overcome financial obstacles to fund PM-SB that apply to other CBOs that implement chronic disease prevention programs. Consistent with interviewee suggestions, there are opportunities for Medicare and Medicaid billing for components of PM-SB and other para-professional delivered mental health and physical disability prevention programs [55]. For example, the Medicare Diabetes Prevention Program (MDPP) is an intervention that targets Medicare beneficiaries that are pre-diabetic to improve their lifestyle and decrease their risk of chronic disease. CBOs that participate in PM-SB and other chronic disease prevention programs are potential suppliers that could receive reimbursement for participants that are Medicare Plan B beneficiaries [55]).

CBOs that work in chronic disease prevention also have an opportunity to engaging local actors, as suggested by staff. The YMCA has a record of supporting chronic disease prevention programs, including MDPP, and has experienced staff such as ETs [56]. Other potential forms of billing are CHW services that can be billed to Medicaid through the 1115 waiver and/or billing of CHWs as providers [57]. Since PM-SB involves direct care with participants and counseling about their health, payment for CHWs services can be processed through a state plan amendment (SPA) [58]. There are resources to train staff on billing third parties for prevention programs such as the National Association of County and City Health Officials’ (NACCHO) billing tool, a virtual tool that trains local agencies on how to bill for services [59].

As the intervention transitions from a research trial to implementation in CBOs, some staff members questioned the need for continuing research assessments if the intervention is already evidence-based, per the trial results. This highlights the “implementation gap” since data collection is often not required at this stage. Yet, being a CBO that implements an EBI but does not collect outcome data can lead to false inferences about the actual impact of an intervention at the population level, an ongoing challenge in the implementation science field [26].

Identifying what data need to be collected for quality assessments and health outcomes without spending large resources requires an important adaptation of PM-SB and other EBIs in CBOs [34, 36, 60]. It will be equally important to define if there will be staff at the CBO that are dedicated to data collection, such as RAs, or if these responsibilities can be integrated into other roles. Similarly, although PM-SB was designed for elder minorities with elevated depression and anxiety symptoms and some deterioration of physical functioning, it will be important to determine if a wider elder minority community, who do not meet all these clinical criteria, can still benefit from the intervention.

We acknowledge several study limitations. Our findings are specific to the PM-SB program. However, our results are like other research that study barriers and strategies to overcome barriers to implement EBIs in CBOs. This is especially salient in the context of chronic disease prevention among the elderly, indicating that they can help inform a broader understanding of the need to include staff’s perspectives when an intervention is transitioning from a research trial to the implementation phase [20, 25, 61]. Most of the data presented comes from a 2-month window from a January–February 2018 meeting. As such, some of the issues that prevailed may have been resolved and others may have emerged in the final stages of the clinical trial. In-depth interviews had a 67% response rate from the sample of participants that completed the survey and focus groups. Since they volunteered to participate in the interview, self-selection could potentially bias responses. However, the interview sample was representative of all the staff roles in the PM-SB study.

## Conclusion

As CBOs continue to become important providers of prevention and intervention programs, they face challenges to ensuring that effective interventions are adopted for implementation and maintained in CBOs once the trial ends. Our study contributes to closing this “implementation gap” by examining staff perceptions of factors that could lead to successful implementation using the ISF. Specifically, we identify factors that facilitate the implementation of this program in the intervention testing phase and suggest strategies for overcoming obstacles. Our findings suggest most barriers are in the delivery system at the organizational level such as: staff-identified turnover, limited time to perform responsibilities, and insufficient funding. Yet, through this study we find viable solutions to strengthen the delivery and support systems to improve the financial health of CBOs and include capacity-building efforts to enhance program implementation. To ensure the best possible transition of an intervention at the clinical trial stage to EBI implementation, we recommend eliciting input from staff to identify barriers and strategies to overcome them, at the individual, organizational, and community level.

## Data Availability

The codebook and data that were analyzed from the survey and transcripts during the current study are available from the corresponding author on reasonable request.

## Abbreviations

CBOs: Community-based organizations
CHW: Community health worker
EBI: Evidence-based interventions
ET: Exercise trainer
ISF: Interactive systems framework for dissemination and implementation
NACCHO: National Association of County and City Health Officials’
PM-SB: Positive minds-strong bodies
RAs: Research assistants
SPA: State plan amendment
